# Comparing medical cannabis use in 5 US states: a retrospective database study

**DOI:** 10.1186/s42238-021-00075-z

**Published:** 2021-05-27

**Authors:** V. Kishan Mahabir, Christopher S. Smith, Christopher Vannabouathong, Jamil J. Merchant, Alisha L. Garibaldi

**Affiliations:** CB2 Insights, 5045 Orbitor Dr, Building 11, Suite 300, Mississauga, ON L4W 4Y4 Canada

**Keywords:** Medical cannabis regulations, Laws, Chronic pain, Anxiety, Post-traumatic stress disorder, State differences

## Abstract

**Background:**

US states have been adopting their own medical cannabis laws since 1996. There is substantial variability in the medical cannabis programs between states, and these differences have not been thoroughly investigated in the literature. The objective of the study was to compare medical cannabis patient characteristics across five states to identify differences potentially caused by differing policies surrounding condition eligibility.

**Methods:**

We conducted secondary analyses following a retrospective study of a registry database with data from 33 medical cannabis evaluation clinics in the US, owned and operated by CB2 Insights. This study narrowed the dataset to include patients from five states with the largest samples: Massachusetts (*n* = 27,892), Colorado (*n* = 16,434), Maine (*n* = 4591), Connecticut (*n* = 2643), and Maryland (*n* = 2403) to conduct an in-depth study of the characteristics of patients accessing medical cannabis in these states, including analysis of variance to compare average ages and number of conditions and chi-squared tests to compare proportions of patient characteristics between states.

**Results:**

Average ages varied between the states, with the youngest average in Connecticut (42.2) and the oldest in Massachusetts (47.0). Males represented approximately 60% of the patients with data on gender in each state. The majority of patients in each state had cannabis experience prior to seeking medical certification. Primary medical conditions varied for each state, with chronic pain, anxiety, and back and neck problems topping the list in varying orders for Massachusetts, Maine, and Maryland. Colorado had 78.7% of patients report chronic pain as their primary condition, and 70.4% of patients in Connecticut reported post-traumatic stress disorder as their primary medical condition.

**Conclusion:**

This study demonstrated the significant impact that policy has on patients’ access to medical cannabis in Massachusetts, Colorado, Maine, Connecticut, and Maryland utilizing real-world data. It highlights how qualifications differ between the five states and brings into question the routes through which patients in states with stricter regulations surrounding eligible conditions choose to seek treatment with cannabis. These patients may turn to alternative treatments, or to the illicit or recreational cannabis markets, where permitted.

**Supplementary Information:**

The online version contains supplementary material available at 10.1186/s42238-021-00075-z.

## Background

Cannabis was added to the United States (US) Controlled Substances Act in 1970, declaring it a Schedule I drug with no accepted medical use and a high potential for abuse (Carliner et al. 2017; Mead 2017). However, there are commonly accepted medicinal effects of cannabis that are largely attributed to delta-9-tetrahydrocannabinol (THC) and cannabidiol (CBD), the two cannabinoids that are understood with the greatest scientific rigour (Pertwee 1997; Pertwee et al. 2010). THC is known for being psychotropic and is the primary concern as regards to cannabis’ potential for abuse (Carliner et al. 2017; Mead 2017), whereas CBD is non-psychotropic and can be accessed legally country-wide when it is derived from hemp with a THC concentration of less than 0.3% dry weight (FDA Regulation of Cannabis and Cannabis-Derived Products, Including Cannabidiol (CBD) 2020; Shannon et al. 2019).

Although the cannabis plant has been used for centuries in traditional medicine, the laws and policies surrounding its use have been drastically shifting in the US over the last two decades (Alsherbiny and Li 2018; Boehnke et al. 2019a; Cambron et al. 2017; Fairman 2016). Though it remains illegal on a federal level, since the mid-1990s, individual states have been developing their own laws for its use; California was the first state to legalize medical cannabis in 1996 (Legislatures NC of S 2020). Other states have been adopting medical cannabis programs since, and as of October 2020, medical cannabis is legal in 33 states and the District of Columbia, with an additional 12 states and the District of Columbia having legalized recreational cannabis (Boehnke et al. 2019a; DISA Global Solutions 2019). Other states have decriminalized the use of recreational cannabis, whereas some still retain a strict prohibitionist approach both medically and recreationally (Legislatures NC of S 2020; Pacula and Smart 2017).

Among the states with medical cannabis programs, the qualifying medical conditions vary widely and are often updated as the base of evidence surrounding cannabis for medical purposes grows (Pacula and Smart 2017). There are also varying levels of reporting requirements among these states, where some have voluntary registries with minimal data included, and others collect detailed information (Boehnke et al. 2019a; Fairman 2016). The variance between states, coupled with the restrictions in place for conducting cannabis research, has made it increasingly difficult to define and characterize the patients using medical cannabis, and the effectiveness of their treatment.

To add to the medical literature and knowledge surrounding the characteristics of patients accessing medical cannabis in the US, we conducted a retrospective study that included 61,379 medical cannabis patients (Mahabir et al. 2020). These data were extracted from a large medical cannabis patient registry created by CB2 Insights that collects data from 33 medical cannabis evaluation clinics across 12 US states. To further the knowledge and understanding of medical cannabis patients at the state-level, we completed an in-depth analysis comparing patients from the five states with the most patients represented in the dataset: Massachusetts, Colorado, Maine, Connecticut, and Maryland. Highlighting the patient differences between the states is important for understanding the effect qualification regulations are having on patients seeking medical cannabis treatment and the potential limitations of these regulations, which may inform states looking to legalize medical cannabis, or those looking to amend current medical cannabis programs. One of the objectives of this study was to identify topics at a state level for further investigation with respect to current medical cannabis programs.

The included states have varying laws surrounding cannabis, summarized in Table 1. Briefly, some states have legalized both medical and recreational cannabis while others have only legalized medical cannabis. States have also handled the legalization of medical cannabis very differently, with some states allowing practitioners to use their medical judgement in qualifying patients for medical cannabis while others require practitioners to adhere to a strictly defined list of eligible conditions. Finally, the states have differed in the timing of their cannabis laws. There are clear differences between the regulations in these states, and the primary objective of this study was to investigate how these regulations, particularly surrounding eligible conditions, may impact the patients who are choosing to seek treatment with medical cannabis. These data were analyzed to answer the following questions:

1. How do key demographic characteristics differ between patients accessing medical cannabis in the five states?
2. How do the primary conditions reported differ between patients in the five states?
3. Are reported primary conditions similar within states with similar qualifying conditions?
4. How does patient income compare to the state median within each state?

**Table 1:** Qualifying conditions for patients seeking medical cannabis in Massachusetts, Colorado, Maine, Connecticut, and Maryland

State	Qualifying condition(s)
**Massachusetts (The 191st General Court of The Commonwealth of Massachusetts** 2020**)** Medically legal: 2012 (late adopter) Recreationally legal: 2016	Debilitating medical conditions: cancer, glaucoma, positive status for human immunodeficiency virus, acquired immune deficiency syndrome (AIDS), hepatitis C, amyotrophic lateral sclerosis (ALS), Crohn’s disease, Parkinson’s disease, multiple sclerosis and other conditions as determined in writing by a qualifying patient’s physician.
**Colorado (Colorado - Official State Web Portal** 2020**)** Medically legal: 2000 (early adopter) Recreationally legal: 2012	*Debilitating medical conditions:* cancer, glaucoma, HIV/AIDS, cachexia, persistent muscle spasms, seizures, severe nausea, and severe (chronic) pain. *Disabling medical conditions*: post-traumatic stress disorder (PTSD), autism spectrum disorder, and any condition for which a physician could prescribe an opioid.
**Maine (128th Maine Legislature** 2020**)** Medically legal: 1999 (early adopter) Recreationally legal: 2016	In the medical provider’s professional opinion, a qualifying patient is likely to receive therapeutic benefit from the medical use of marijuana to treat or alleviate the patient’s debilitating medical condition.
**Connecticut (Connecticut State Department of Consumer Protection** 2020**)** Medically legal: 2012 (late adopter) Decriminalized: 2011	*Adult medical conditions:* cancer, glaucoma, HIV/AIDS, Parkinson’s disease, multiple sclerosis, damage to nervous tissue of the spinal cord with objective neurological indication of intractable spasticity, epilepsy, cachexia, wasting syndrome, Crohn’s disease, post-traumatic stress disorder, sickle cell disease, post laminectomy syndrome with chronic radiculopathy, severe psoriasis and psoriatic arthritis, amyotrophic lateral sclerosis, ulcerative colitis, complex regional pain syndrome Type 1 and Type II, cerebral palsy, cystic fibrosis, irreversible spinal cord injury with objective neurological indication of intractable spasticity, terminal illness requiring end-of-life care, uncontrolled intractable seizure disorder, spasticity or neuropathic pain associated with fibromyalgia, severe rheumatoid arthritis, post herpetic neuralgia, hydrocephalus with intractable headache, intractable headache syndromes, neuropathic facial pain, muscular dystrophy, osteogenesis imperfecta, chronic neuropathic pain associated with degenerative spinal disorders, interstitial cystitis, MALS Syndrome (median arcuate ligament syndrome), vulvodynia and vulvar burning, intractable neuropathic pain that is unresponsive to standard medical treatments, and Tourette syndrome.
**Maryland (Natalie** n.d.**)** Medically legal: 2014 (late adopter) Decriminalized: 2014	Cachexia, anorexia, wasting syndrome, severe or chronic pain, severe nausea, seizures, severe or persistent muscle spasms, glaucoma, post-traumatic stress disorder (PTSD), or another chronic medical condition which is severe and for which other treatments have been ineffective.

Table 1 summarizes the medical cannabis regulations for each state, current as of August 2020, as well as the year legalization laws were passed for medical cannabis and recreational cannabis

## Methods

This was a retrospective database study of patients seeking medical cannabis certification in the US. Data were extracted from the database software utilized in CB2 Insights’ clinical network. CB2 Insights operates one of the largest collections of medical cannabis evaluation clinics in the US, collectively assessing approximately 100,000 patients per year seeking access to medical cannabis, using a single and consistent software that contributes data to a patient registry. These 33 independent clinics are not connected to dispensaries or producers of medical cannabis and are situated across 12 states (number of clinics): Colorado (6), Connecticut (1), Delaware (2), Illinois (1), Maine (1), Maryland (1), Massachusetts (10), Missouri (1), New Jersey (5), New York (1), Rhode Island (2), and Pennsylvania (2), Colorado (6), Connecticut (1), Delaware (2), Illinois (1), Maine (1), Maryland (1), Massachusetts (10),Missouri (1), New Jersey (5), New York (1), Rhode Island (2), and Pennsylvania (2). Patients access these clinics by physician-referral or self-referral through word of mouth, community out-reach, and marketing. Over 95% of data were collected via face-to-face interview, with the remaining collected via telemedicine. Patients presenting to any of the clinics are required to complete the same baseline information upon intake, including demographic, medical, and therapeutic information; however, certain characteristics such as race and gender were not made mandatory initially, and are not reported for all patients. Baseline questions include patient-reported tobacco smoking and alcohol use, current or past substance abuse of drugs and/or alcohol, use of illicit (illegal) drugs, medication use, and non-pharmacologic therapies. Medication use is an open-ended question that may be completed by transcribing a medication list into the software, which leaves room for errors and may be a limitation of the data. All patients indicate their primary reason for seeking access to medical cannabis (their qualifying condition) and are asked to report all comorbid conditions for which they are also seeking medical cannabis. Patients are required to provide supporting documentation of their medical histories and relevant conditions for review and verification, in the form of medical records or a letter from another physician. Review of medical documentation, in combination with a medical evaluation by a state-authorized physician or nurse practitioner, is used to confirm their qualification for medical cannabis within their respective state.

Advarra Institutional Review Board (IRB) reviewed the protocol for these publications and determined it to be exempt from IRB oversight (Pro00042652) as the study had minimal risk, patient identifiers were not included in data exports, and it did not require direct patient contact. Data were exported for 62,145 patients who were seen for their initial assessment between November 18, 2018 (when the technology and standardized protocol were introduced into the clinics) and March 18, 2020. Data were exported without any patient identifiers to ensure patient anonymity. Eligibility criteria were applied to the dataset, and the following patients were removed: 1) 77 patients without a valid date of birth; 2) 78 patients younger than 18; and 3) 611 patients without a primary medical condition reported. To further investigate the differences in demographic, socioeconomic, and medical characteristics of patients accessing medical cannabis in different states, we narrowed our dataset to include the five states with the largest number of registry patients. Patients from Massachusetts (Burlington, Danvers, Fall Rivers, Northampton, Pittsfield, Seekonk, Stoughton, Waltham, Worcester, Yarmouth Port), Colorado (Broomfield, Colorado Springs, Denver, Pueblo), Maine (Bangor), Connecticut (Hartford), and Maryland (Columbia, Baltimore) remained in the dataset for further analysis, and patients from Delaware, Illinois, Missouri, New Jersey, New York, Rhode Island, and Pennsylvania were removed.

Data from the database software utilized in CB2 Insights’ clinical network were also merged with US tax data, which provides tabulations of income tax data by ZIP code in order to estimate household income based on individual patients’ ZIP codes. Median household income values from the 2018 dataset purchased from Cubit Planning Inc. were used (US Income Statistics 2020). Cubit Planning Inc. summarizes the most current income statistics from the US Census Bureau.

States were classified as early medical cannabis adopters (2000 and earlier) (Maine (1999) and Colorado (2000)) or late adopters (Connecticut (2012), Massachusetts (2012), and Maryland (2014)), recreationally legal (Colorado, Massachusetts, and Maine), or decriminalized (Connecticut and Maryland), as summarized in Table 1.

The final dataset was analyzed using RStudio (Boston, MA). The analyses were completed to investigate differences in demographic, socioeconomic, and medical characteristics of patients between the five states. Descriptive statistics, expressed as a mean (standard deviation (SD)) or median (interquartile range (IQR)), were used as appropriate for continuous variables, and number (percent) for categorical variables to summarize information. *T* tests were conducted when comparing means between two groups, chi-squared tests when comparing proportions, and an analysis of variance (ANOVAs) to compare means of more than two groups using Tukey’s honestly significant difference post-hoc test. All tests were completed with a significance level of 0.05. *P* values less than 0.001 were expressed as *p* < 0.001.

## Results

### Demographic characteristics

There were 61,379 patients included in the original analysis. For the purposes of this study, patients from nine states were removed, leaving 53,963 patients from five states: Massachusetts (*n* = 27,892), Colorado (*n* = 16,434), Maine (*n* = 4591), Connecticut (*n* = 2643), and Maryland (*n* = 2403) (Supplemental Material, Figure 1). The average age across the five states varied significantly (*p* < 0.001) (Table 2). The youngest average age was reported in Connecticut (42.2, SD = 14.4) and the oldest in Massachusetts (47.0, SD = 15.7). Males represented approximately 60% of the patients across the entire sample of patients. The proportion of males in Colorado and Maine were significantly larger than the other states (*p* < 0.001) at 61.1% and 60.9%, respectively, compared to proportions in Connecticut, Massachusetts, and Maryland at 58.5%, 58.4%, and 58.8%, respectively. The average age of females was older than males in each state, but the difference was only significant in Colorado, Massachusetts, and Maine (45.1 vs 42.2, *p* < 0.001; 48.9 vs 46.2, *p* < 0.001; and 47.0 vs 45.6, *p* = 0.0036, respectively). Of the patients with race information reported, White individuals represented the majority in each state, ranging from 68.2% in Maryland to 96.1% in Maine. There were significant differences in reported tobacco smoking, alcohol use, and substance abuse between states (*p* < 0.001). Smoking was lowest in Maryland (11.3%) and highest in Colorado (24.7%). Alcohol use ranged from 38.5% in Colorado to 44.7% in Massachusetts. History of substance abuse was lowest in Colorado (2.3%) and highest in Connecticut (12.5%).

**Table 2:** Sociodemographic and medical characteristics of 53,963 patients seeking medical cannabis certification from CB2 Insights clinics in five states

Characteristic	Massachusetts	Colorado	Maine	Connecticut	Maryland	Total
	***n*** = 27,892 mean (SD) or ***n*** (%)	***n*** = 16,434 mean (SD) or ***n*** (%)	***n*** = 4591 mean (SD) or ***n*** (%)	***n*** = 2643 mean (SD) or ***n*** (%)	***n*** = 2403 mean (SD) or ***n*** (%)	***n*** = 61,379 mean (SD) or %
**Age** (years), mean (SD)^a,b^	47.0 (15.9)	43.3 (15.9)	46.2 (15.6)	42.2 (14.4)	45.7 (15.7)	45.5 (15.8)
**Gender**						
Male	14,615 (52.4%)	9,969 (60.7%)	2,720 (59.2%)	1,506 (57.0%)	1,019 (42.4%)	54.8%
Female	10,413 (37.3%)	6,353 (38.7%)	1,746 (38.0%)	1,067 (40.4%)	714 (29.7%)	37.8%
Non-binary	12 (0.0%)	5 (0.0%)	1 (0.0%)	6 (0.2%)	4 (0.2%)	0.1%
Unknown/unspecified	2,851 (10.2%)	107 (0.7%)	124 (2.7%)	64 (2.4%)	666 (27.7%)	7.3%
**Race**	*n=11,005*	*n=12,266*	*n=1,452*	*n=1,927*	*n=654*	*n=32,275*
White	10,304 (93.6%)	10,372 (84.6%)	1,396 (96.1%)	1,615 (83.8%)	446 (68.2%)	87.5%
Black	451 (4.1%)	1,169 (9.5%)	17 (1.2%)	268 (13.9%)	184 (28.1%)	8.5%
Other	110 (1.0%)	316 (2.6%)	20 (1.4%)	21 (1.1%)	6 (0.9%)	1.6%
Asian	94 (0.9%)	117 (1.0%)	7 (0.5%)	13 (0.7%)	10 (1.5%)	0.9%
American Indian/Alaska Native	9 (0.1%)	210 (1.7%)	10 (0.7%)	6 (0.3%)	2 (0.3%)	0.8%
Middle Eastern	22 (0.2%)	19 (0.2%)	0 (0.0%)	3 (0.2%)	3 (0.5%)	0.2%
Native Hawaiian/other Pacific Islander	2 (0.0%)	47 (0.4%)	2 (0.1%)	0 (0.0%)	2 (0.3%)	0.2%
South East Asian	13 (0.1%)	16 (0.1%)	0 (0.0%)	1 (0.1%)	1 (0.2%)	0.1%
**Surrogate household income**	*n=25,182*	*n=15,627*	*n=4,222*	*n=2,595*	*n=1,718*	*n=56,083*
Median	$75,480	$64,251	$55,737	$71,961	$84,257	$69,481
(IQR)	($37,785)	($32,120)	($19,633)	($29,852)	($37,917)	($35,807)
**Smoking status**^a,b^						
Smoker	3,994 (14.3%)	4,059 (24.7%)	1,022 (22.3%)	638 (24.1%)	271 (11.3%)	18.8%
Non-Smoker	23,897 (85.7%)	12,375 (75.3%)	3,569 (77.7%)	2,005 (75.9%)	2,132 (88.7%)	81.2%
**Alcohol consumption**^a,b^						
Yes	12,459 (44.7%)	6,323 (38.5%)	1,905 (41.5%)	1,081 (40.9%)	976 (40.6%)	42.5%
No	15,432 (55.3%)	10,111 (61.5%)	2,686 (58.5%)	1,562 (59.1%)	1,427 (59.4%)	57.5%
**Previous cannabis experience**^a,b^						
Yes	20,118 (72.1%)	10,450 (63.6%)	3,509 (76.4%)	1,959 (74.1%)	1,399 (58.2%)	66.9%
No	7,773 (27.9%)	5,984 (36.4%)	1,082 (23.6%)	684 (25.9%)	1,004 (41.8%)	33.1%
**Use of non-cannabis illicit drugs**						
Yes	95 (0.3%)	89 (0.5%)	15 (0.3%)	12 (0.5%)	5 (0.2%)	0.4%
No	27,796 (99.7%)	16,345 (99.5%)	4,576 (99.7%)	2,631 (99.5%)	2,398 (99.8%)	99.6%
**History of substance abuse**^a,b^						
Yes	1,875 (6.7%)	380 (2.3%)	296 (6.4%)	330 (12.5%)	81 (3.4%)	5.6%
No	26.016 (93.3%)	16,054 (97.7%)	4,295 (93.6%)	2,313 (87.5%)	2,322 (96.6%)	94.4%
**Number of medications**^a,b^						
0	14,272 (51.2%)	11,233 (68.4%)	2,625 (57.2%)	1,252 (47.4%)	1,216 (50.6%)	55.8%
1	3,780 (13.6%)	1,601 (9.7%)	569 (12.4%)	358 (13.5%)	427 (17.8%)	12.8%
2	2,263 (8.1%)	956 (5.8%)	399 (8.7%)	210 (8.0%)	226 (9.4%)	7.7%
3	1,683 (6.0%)	738 (4.5%)	244 (5.3%)	179 (6.8%)	158 (6.6%)	5.7%
4	1,115 (4.0%)	515 (3.1%)	167 (3.6%)	120 (4.5%)	124 (5.1%)	3.9%
5+	4,779 (17.1%)	1,391 (8.5%)	587 (12.8%)	524 (19.8%)	252 (10.5%)	14.0%
**Average number of medications**	2.2 (3.5)	1.2 (2.5)	1.7 (3.1)	2.5 (3.7)	1.7 (2.8)	2.1 (4.8)
**Number of non-pharmacologic therapies used**						
0	10,408 (37.3%)	7,979 (48.6%)	1,570 (34.2%)	997 (37.7%)	1,065 (44.3%)	40.6%
1	5,558 (19.9%)	3,537 (21.5%)	1,121 (24.4%)	586 (22.2%)	487 (20.3%)	20.9%
2	4,461 (16.0%)	2,256 (13.7%)	789 (17.2%)	388 (14.7%)	322 (13.4%)	15.3%
3	3,184 (11.4%)	1,264 (7.7%)	503 (11.0%)	265 (10.0%)	264 (11.0%)	10.1%
4	1,904 (6.9%)	674 (4.1%)	273 (5.9%)	181 (6.8%)	148 (6.2%)	6.0%
5+	2,377 (8.5%)	724 (4.4%)	335 (7.3%)	226 (8.6%)	117 (4.9%)	7.1%
**Non-pharmacologic therapies**						
Exercise	12,540 (45.0%)	5,935 (36.1%)	2,151 (46.9%)	1,137 (43.0%)	935 (38.9%)	42.1%
Massage therapy	6,750 (24.2%)	2,937 (17.9%)	972 (21.2%)	511 (19.3%)	466 (19.4%)	21.6%
Chiropractor	5,540 (19.9%)	2,272 (13.8%)	1,050 (22.9%)	362 (13.7%)	349 (14.5%)	18.0%
Mental health counselling	6,117 (21.9%)	1,579 (9.6%)	864 (18.8%)	757 (28.6%)	421 (17.5%)	18.3%
Physiotherapy	1,342 (4.8%)	1,270 (7.7%)	215 (4.7%)	102 (3.9%)	100 (4.2%)	5.5%
Mindfulness-based cognitive therapy	2,760 (9.9%)	1,111 (6.8%)	251 (5.5%)	295 (11.2%)	175 (7.3%)	8.4%
Aromatherapy	1,669 (6.0%)	1,015 (6.2%)	293 (6.4%)	211 (8.0%)	139 (5.8%)	6.3%
Homeopathic medicine	1,467 (5.3%)	793 (4.8%)	278 (6.1%)	123 (4.7%)	92 (3.8%)	5.2%
Acupuncture	3,309 (11.9%)	769 (4.7%)	503 (11.0%)	206 (7.8%)	243 (10.1%)	9.2%
Cognitive behavioural therapy (CBT)	1,885 (6.8%)	478 (2.9%)	270 (5.9%)	213 (8.1%)	115 (4.8%)	5.7%
Naturopathic medicine	860 (3.1%)	403 (2.5%)	149 (3.2%)	104 (3.9%)	49 (2.0%)	2.9%
Reiki	1,380 (4.9%)	308 (1.9%)	205 (4.5%)	113 (4.3%)	71 (3.0%)	3.8%
Addictions counselling	602 (2.2%)	137 (0.8%)	131 (2.9%)	115 (4.4%)	35 (1.5%)	1.9%
Acudetox	19 (0.1%)	29 (0.2%)	7 (0.2%)	4 (0.2%)	6 (0.2%)	0.1%
Other	42 (0.1%)	11 (0.1%)	18 (0.4%)	8 (0.3%)	8 (0.3%)	0.4%
**Number of comorbid conditions**						
0	2,206 (7.9%)	6,263 (38.1%)	294 (6.4%)	212 (8.0%)	213 (8.9%)	17.6%
1	5,719 (20.5%)	5,548 (33.8%)	940 (20.5%)	429 (16.2%)	663 (27.6%)	26.0%
2	4,935 (17.7%)	2,540 (15.5%)	991 (21.6%)	402 (15.2%)	459 (19.1%)	16.1%
3	4,028 (14.4%)	1,183 (7.2%)	700 (15.2%)	370 (14.0%)	415 (17.3%)	11.9%
4	3,276 (11.8%)	548 (3.3%)	531 (11.6%)	310 (11.7%)	253 (10.5%)	9.0%
5+	7,728 (27.7%)	352 (2.1%)	1,135 (24.7%)	920 (34.9%)	400 (16.6%)	19.4%
**Total number of conditions,** mean (SD)^a,b^	4.3 (2.8)	2.1 (1.3)	4.1 (2.6)	4.8 (3.1)	3.6 (2.1)	3.7 (2.6)

Table 2 summarizes key patient characteristics of patients seeking medical cannabis access at CB2 Insights clinics between November 2018 and March 2020 across five states. The total column includes all 61,379 patients included in the initial study for comparison, including patients from 12 states (Mahabir et al. 2020). Total number of conditions refers to a count of the number of comorbid conditions plus one for the patients’ primary condition. SD = standard deviation

^a^Indicates that statistical test for significance was completed between the five states; the total numbers were not included in statistical tests. ANOVA was conducted for average age and average number of conditions between states. Chi-squared tests were conducted for differences in proportions between states

^b^Indicates that statistical test was significant, and the characteristic was significantly different between states

The median income varied between the states but trended towards the median income of each state. Patients in Maryland and Maine had household incomes very similar to the state median ($84,257 compared to $83,242 and $55,737 compared to $55,602, respectively), whereas the median patient household incomes in Colorado, Connecticut, and Massachusetts were lower than the state median ($64,251 compared to $71,953, $71,961 compared to $76,348 and $75,480 compared to $79,835, respectively) (Guzman 2020). State income distributions compared to sample patient income distributions are compared in Supplemental Materials Figures 2–6. Each individual state sample followed a distribution similar to the overall state.

### Prior cannabis experience

The majority of patients in each state had cannabis experience prior to seeking medical certification. Maine reported the highest proportion of patients with prior experience at 76.4%, and Maryland reported the lowest at 58.2%. Prior experience in Connecticut, Massachusetts, and Colorado was reported at 74.1%, 72.1%, and 63.6%, respectively. States that were early adopters of medical cannabis had a significantly lower proportion of patients with previous cannabis experience than states that were late adopters (66.4% compared to 71.3%, *p* < 0.001). States that have legalized recreational cannabis reported a higher percentage of patients with prior experience (69.7% compared to 66.5%, *p* < 0.001).

### Medication and non-pharmacologic therapy information

Connecticut had the largest proportion of patients who reported using at least one prescription medication (52.6%), whereas Colorado had significantly less (31.6%, *p* < 0.001). Similarly, Colorado reported the smallest proportion (8.5%) of patients taking five or more medications, and Connecticut reported the largest (19.8%). All states had at least 50% of patients who reported using a non-pharmacologic therapy. Exercise was the most commonly reported therapy in all states (36.1 to 46.9%), followed by massage therapy in Massachusetts, Colorado, and Maryland (24.2%, 17.9%, and 19.4%, respectively), chiropractor in Maine (22.9%), and mental health counselling in Connecticut (28.6%).

### Qualifying medical conditions and comorbid conditions

**Fig. 1:**
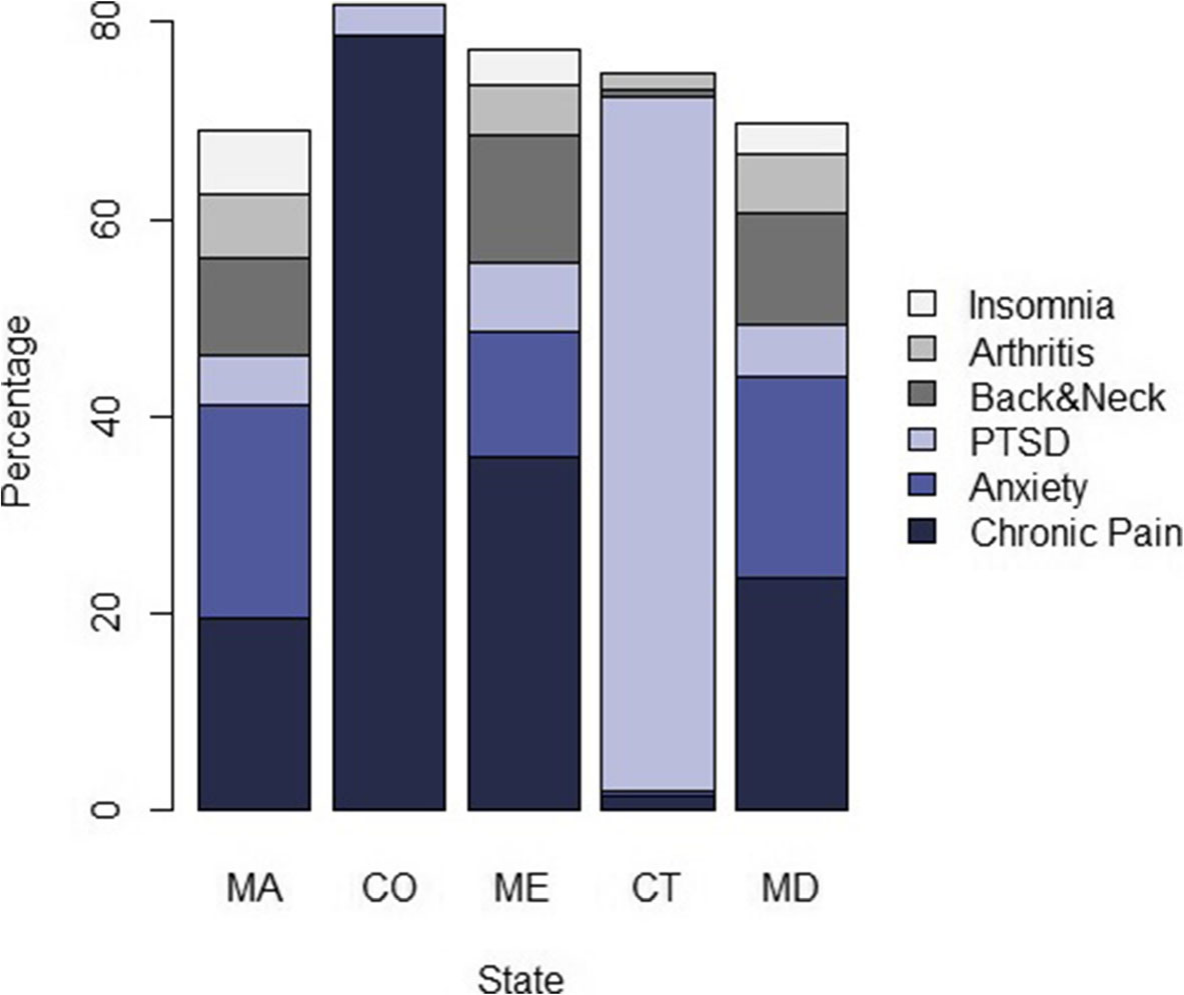
Top 6 primary qualifying conditions reported by medical cannabis patients, by state. PTSD = post-traumatic stress disorder; MA = Massachusetts; CO = Colorado; ME = Maine; CT = Connecticut; MD = Maryland

The reported primary conditions of patients varied widely between states (Table 3). Massachusetts, Maine, and Maryland had a similar spread of proportions of primary medical conditions, with 5 or 6 conditions in each state being reported by 5% or more of patients (Fig. 1). In each of these states, chronic pain (19.4%, 35.9%, 23.7%), anxiety (21.7%, 12.7%, 20.3%), and back and neck problems (9.8%, 12.9%, 11.4%) were the top 3 medical conditions reported, although the order varied in each state. The distribution of proportions in Colorado and Connecticut was significantly different than the former three states. In Colorado, 78.7% of patients reported chronic pain as their primary medical condition, followed by muscle spasms at 4.7%. In Connecticut, 70.4% of patients reported PTSD as their primary medical condition, with spinal cord injury/disease reported second most often (7.3%).

**Table 3:** Summary of primary and comorbid conditions reported by 53,963 patients seeking medical cannabis certification across five states

Condition	Massachusetts ***n*** = 27,892 ***n*** (%)		Colorado ***n*** = 16,434 ***n*** (%)		Maine ***n*** = 4591 ***n*** (%)		Connecticut ***n*** = 2643 ***n*** (%)		Maryland ***n*** = 2403 ***n*** (%)		Total ***n*** = 61,379 %
**Primary condition**											
Chronic pain	5407	(19.4%)	12926	(78.7%)	1646	(35.9%)	42	(1.6%)	569	(23.7%)	38.8%
Anxiety	6062	(21.7%)	5	(<0.1%)	581	(12.7%)	9	(0.3%)	487	(20.3%)	13.5%
Post-traumatic stress disorder	1439	(5.2%)	456	(2.8%)	323	(7.0%)	1860	(70.4%)	126	(5.2%)	8.4%
Back and neck problems	2737	(9.8%)	22	(0.1%)	590	(12.9%)	24	(0.9%)	273	(11.4%)	6.5%
Arthritis	1785	(6.4%)	14	(0.1%)	232	(5.1%)	44	(1.7%)	142	(5.9%)	3.9%
Insomnia	1811	(6.5%)	1	(0.0%)	169	(3.7%)	0	(0.0%)	76	(3.2%)	3.4%
Cancer-related pain	752	(2.7%)	447	(2.7%)	84	(1.8%)	98	(3.7%)	48	(2.0%)	2.7%
Depression	1050	(3.8%)	0	(0.0%)	58	(1.3%)	0	(0.0%)	102	(4.2%)	2.0%
Migraines	810	(2.9%)	26	(0.2%)	136	(3.0%)	36	(1.4%)	54	(2.2%)	2.0%
Muscle spasms	148	(0.5%)	780	(4.7%)	32	(0.7%)	0	(0.0%)	26	(1.1%)	1.7%
ADD/ADHD	866	(3.1%)	0	(0.0%)	71	(1.5%)	0	(0.0%)	39	(1.6%)	1.6%
Chronic nausea	248	(0.9%)	546	(3.3%)	60	(1.3%)	0	(0.0%)	16	(0.7%)	1.5%
Fibromyalgia	448	(1.6%)	14	(0.1%)	57	(1.2%)	53	(2.0%)	28	(1.2%)	1.2%
Headaches	292	(1.0%)	242	(1.5%)	94	(2.0%)	29	(1.1%)	15	(0.6%)	1.2%
Spinal cord injury/disease	263	(0.9%)	0	(0.0%)	15	(0.3%)	192	(7.3%)	22	(0.9%)	0.9%
Other	3774	(13.5%)	955	(5.8%)	443	(9.6%)	256	(9.7%)	380	(15.8%)	10.7%
**Comorbid Conditions**											
Anxiety	9815	(35.2%)	1226	(7.5%)	1543	(33.6%)	1534	(58.0%)	710	(29.5%)	28.3%
Back and neck problems	7858	(28.2%)	1642	(10.0%)	1323	(28.8%)	807	(30.5%)	564	(23.5%)	23.7%
Depression	8290	(29.7%)	481	(2.9%)	1059	(23.1%)	1014	(38.4%)	560	(23.3%)	21.9%
Insomnia	9442	(33.9%)	740	(4.5%)	1157	(25.2%)	782	(29.6%)	491	(20.4%)	23.2%
Chronic pain	6227	(22.3%)	1072	(6.5%)	1314	(28.6%)	653	(24.7%)	584	(24.3%)	18.2%
Stress	7171	(25.7%)	73	(0.4%)	913	(19.9%)	947	(35.8%)	571	(23.8%)	11.2%
Headaches	4569	(16.4%)	1339	(8.1%)	756	(16.5%)	487	(18.4%)	277	(11.5%)	14.2%
Arthritis	5013	(18.0%)	700	(4.3%)	767	(16.7%)	452	(17.1%)	286	(11.9%)	14.0%
Muscle spasms	3234	(11.6%)	2141	(13.0%)	668	(14.6%)	331	(12.5%)	277	(11.5%)	12.8%
Migraines	3508	(12.6%)	692	(4.2%)	392	(8.5%)	362	(13.7%)	189	(7.9%)	9.9%
Post-traumatic stress disorder	3265	(11.7%)	1257	(7.6%)	618	(13.5%)	114	(4.3%)	201	(8.4%)	10.0%
ADD/ADHD	2855	(10.2%)	88	(0.5%)	366	(8.0%)	377	(14.3%)	216	(9.0%)	7.5%
Mood disorders	2222	(8.0%)	30	(0.2%)	261	(5.7%)	308	(11.7%)	140	(5.8%)	6.0%
Chronic nausea	1544	(5.5%)	1665	(10.1%)	350	(7.6%)	0	(0.0%)	77	(3.2%)	7.2%
Neuropathic pain	1660	(6.0%)	165	(1.0%)	253	(5.5%)	200	(7.6%)	96	(4.0%)	4.8%
Irritable bowel syndrome	1698	(6.1%)	82	(0.5%)	292	(6.4%)	197	(7.5%)	83	(3.5%)	4.6%
Appetite stimulation	1875	(6.7%)	112	(0.7%)	256	(5.6%)	157	(5.9%)	102	(4.2%)	4.7%
Fibromyalgia	967	(3.5%)	189	(1.2%)	173	(3.8%)	83	(3.1%)	48	(2.0%)	2.9%
Spinal cord injury	928	(3.3%)	27	(0.2%)	131	(2.9%)	105	(4.0%)	63	(2.6%)	2.7%
Obsessive compulsive behavior	904	(3.2%)	11	(0.1%)	89	(1.9%)	132	(5.0%)	45	(1.9%)	2.3%

Table 3 provides a summary of the reported primary and comorbid conditions of patients seeking medical cannabis access at CB2 Insights clinics between November 2018 and March 2020 across five states. The total column includes all 61,379 patients included in the initial study for comparison, including patients from 12 states (Mahabir et al. 2020). Patients could only report one primary condition. All conditions that represented at least 2.0% of the sample in one state are listed, the remaining are grouped under “other”. Patients could report multiple comorbid conditions, but not the same comorbid condition as their primary. The top 20 are reported in the table. ADD = attention deficit disorder; ADHD = attention deficit hyperactivity disorder

Anxiety was the most commonly reported comorbid condition in Massachusetts, Maine, Connecticut, and Maryland (35.2%, 33.6%, 58.0%, 29.5%). Back and neck problems, depression, insomnia, chronic pain, and stress were also all reported by at least 20% of patients in each of these four states as well. Fewer than 10% of patients in each of these four states did not report having a comorbid condition. Contrarily, almost 40% of patients in Colorado did not report a comorbid condition and, of those who did, muscle spasms were reported by most patients (13.0%), followed by chronic nausea (10.1%) and back and neck problems (10.0%).

The total number of conditions reported varied significantly between states (*p* < 0.001) (Table 2). Patients in Connecticut reported the highest number of average conditions (4.8, SD = 3.1), whereas patients in Colorado reported the lowest (2.1, SD = 1.3).

## Discussion

This retrospective study provided a detailed description of cannabis users across five US states: Massachusetts, Colorado, Maine, Connecticut, and Maryland. Patient demographics as well as medical characteristics varied significantly between the states. Males consistently represented a higher proportion of patients in each state, and females were on average older than males within each state, but this difference was not significant in Connecticut or Maryland. At a high level, relatively similar findings have been reported in other studies, with males accessing medical cannabis more than females, and an average age of patients in their forties (Eurich et al. 2019; Fairman 2016; Piper et al. 2017a; Sexton et al. 2016).

Reviewing the race breakdown of patients accessing medical cannabis at our clinics in each state, the distributions were similar when compared to the overall racial breakdown within each state overall, with some variation. White individuals were slightly underrepresented in Connecticut and Colorado, and overrepresented in Maryland and Massachusetts (U.S. Census Bureau QuickFacts: United States 2020). Black individuals were adequately represented when compared to state demographics in Connecticut, Maine, and Maryland, but underrepresented in Massachusetts and overrepresented in Colorado. We hypothesize that the varying representation in Massachusetts and Colorado is due specifically to the locations of the clinics, as they are in areas with a proportion of Black individuals lower than the state average in Massachusetts, and higher in Colorado. This state-by-state breakdown gives an interesting perspective on race and medical cannabis use. White individuals are often the largest race group reported accessing medical cannabis in studies; however, this study demonstrates that race representation may be proportional to that within the jurisdiction.

The median estimated household incomes for patients in the registry from Massachusetts, Colorado, and Connecticut were lower than the state medians (Bureau UC. U.S n.d.), which is in line with similar studies that report that medical cannabis users tend to have a lower income than the median (Reiman 2007; Sexton et al. 2016). Contrarily, patients in Maryland and Maine had estimated household incomes very similar to the state median. It is important to highlight how reporting income as a whole for this sample would have skewed these results drastically: the median household income of the sample is $68,874, which is higher than the US median of $61,937 (Bureau UC. U.S n.d.). Reporting by state gives a much clearer picture of the sociodemographic characteristics of medical cannabis users. Further, distributions for each sample roughly mirrored the state-level distribution, indicating that income may not be a contributing factor to patients seeking medical cannabis.

All states reported a higher percentage of patients with prior experience than the US average for lifetime cannabis use, which was 45.3% in 2018 (2018 NSDUH Detailed Tables | CBHSQ Data 2020). We hypothesized that prior experience would be higher in states that were early adopters of medical cannabis, but the study results did not support this. Stigma may be lower in states where medical cannabis has been legal for a substantial period of time, leading to inexperienced patients being more willing to seek proper medical treatment with cannabis initially, rather than self-treating. However, prior use was higher in states with legalized recreational cannabis, although the proportions varied between these states. This finding is contrary to findings that legalizing cannabis does not necessarily increase use, although the evidence surrounding this is inconsistent (Cerdá et al. 2020; Cerdá et al. 2017; Cerdá et al. 2011; Gorman and Huber 2007; Leyton 2019; Marijuana Decriminalization and Its Impact on Use - NORML - Working to Reform Marijuana Laws 2020). The process by which patients choose to seek treatment with medical cannabis merits further investigation, as an understanding of this may help encourage patients who would like to use cannabis medically to seek guidance and oversight by a medical team as their first option.

Following our first study in which chronic pain was the clear frontrunner for patients’ primary medical condition, the same result was expected when looking at individual states, but this was not the case. The impact of the differences between state qualifying conditions is highlighted well by our results. In Massachusetts, Maine, and Maryland, the distributions of primary conditions were similar. Within each of these states guidelines’ for eligible qualifying conditions, patients with other conditions for which the practitioner believes medical cannabis may be an effective treatment may be granted certification (128th Maine Legislature 2020; Natalie n.d.; The 191st General Court of The Commonwealth of Massachusetts 2020). Massachusetts and Maryland have a short list of medical conditions included with the aforementioned statement, whereas Maine does not provide a list (Table 1) (128th Maine Legislature 2020; Natalie n.d.; The 191st General Court of The Commonwealth of Massachusetts 2020). Interestingly, none of the top three primary medical conditions reported in Massachusetts or Maryland (chronic pain, anxiety, back and neck problems) appear on the short lists. We hypothesize that allowing practitioners to use their medical judgement when qualifying patients may provide patients greater access to medical cannabis within these states and may provide a clearer picture of the true conditions for which patients seek medical cannabis. The breakdown of primary medical conditions reported in Colorado and Connecticut followed much different patterns, and we again hypothesize that this is a direct result of the state-defined qualifying conditions.

In Colorado, patients may qualify based on a limited number of conditions, or any condition for which a physician could prescribe an opioid (Colorado - Official State Web Portal 2020). The latter is heavily represented in the patient sample from Colorado, as almost 80% of patients reported chronic pain as their primary medical condition (Boehnke et al. 2016; Boehnke et al. 2019b; Lucas et al. 2019; Piper et al. 2017b). The medical conditions with the highest proportion of patients following chronic pain were muscle spasms and chronic nausea, which are explicitly stated in the guidelines to be qualifying conditions.

Connecticut has a lengthy list of qualifying conditions, but despite this list, the majority of patients reported PTSD as their primary medical condition (Connecticut State Department of Consumer Protection 2020). There is not a clear indication as to why PTSD is reported by the majority of patients accessing medical cannabis in Connecticut, compared to the other states in which 10–20% of patients reported PTSD as a primary or comorbid condition, which aligns with an article indicating that 23% of patients seeking medical cannabis for the first time screen positive for lifetime PTSD (Bohnert et al. 2014). We can, however, hypothesize that the high proportion of PTSD patients may be due to the proximity of the clinics to a military base. Prevalence of PTSD among veterans is often reported to be greater than the general population, with an estimate that 11 to 20% of Iraq and Afghanistan veterans suffer from PTSD, whether diagnosed or not, compared to roughly 2% of the general US population (Ghaffarzadegan et al. 2016). However, the use of cannabis is strictly prohibited among active-duty military and, even among veterans, its use is discouraged (VA and Marijuana – what veterans need to know - Public Health 2020).

In Massachusetts, Maine, Connecticut, and Maryland, over 90% of patients reported at least one comorbid condition, whereas in Colorado the same was reported by just over 60% of patients. Among the comorbid conditions reported in the former four states, they were similar to results from a survey that included patients from across the US, Canada, and the UK, where anxiety, back and neck problems, depression, insomnia, chronic pain, stress, and headaches were among the highest reported conditions (Sexton et al. 2016). It is interesting to note that in Connecticut a large number of patients reported comorbid conditions that are not a qualifying condition for the state, demonstrating that although these conditions do not qualify them they are still able to use medical cannabis as a treatment for them as their primary condition is eligible. The same trend is seen within Colorado, but with fewer patients. Patients in Connecticut reported the highest average number of comorbid conditions, and Colorado reported the lowest. We can only speculate here what the source of these differences are between states, but it may be related to the attitudes surrounding medical cannabis, with patients in Colorado not feeling the need to strongly advocate and justify their need for medical cannabis by listing numerous conditions. However, this could also be explained by differing practices for completing assessment appointments between the clinics.

Strengths of this study include the large sample size and detailed information on the patients in these five states. All data collection was verified by a qualified practitioner at the time of input, and thorough information was available on patients’ medical conditions. Limitations include missing data on race and gender, a lack of ethnicity information, the use of surrogate income data, and the absence of data on patients who did not qualify for medical cannabis certification and were not included in the registry. Another limitation is that the data came from a single network of clinics and may not be representative of all medical cannabis patients in these states. According to the Marijuana Policy Project website, Massachusetts has 69,008 registered medical cannabis patients (as of March 2020), Colorado has 81,722 (as of March 2020), Maine has 65,368 (as of December 2019), Connecticut has 41,212 (as of May 2020), and Maryland has 99,912 (as of May 2020). Our largest representation is in Massachusetts at approximately 40%, followed by approximately 20% in Colorado, 7% in Maine, 6% in Connecticut, and 2% in Maryland (Medical Marijuana Patient Numbers 2021).

## Conclusions

To our knowledge, this study was the first to offer detailed, state-level insights into the characteristics of patients accessing medical cannabis. It highlights the impact of the differing state-eligible qualifying conditions between the five states of Massachusetts, Colorado, Maine, Connecticut, and Maryland and brings into question the routes through which patients choose to seek treatment with cannabis in states where they may not qualify based on their condition. These patients may turn to alternative treatments, or to the illicit or recreational cannabis markets, where permitted. Alternatively, patients may choose to misrepresent their condition to fit in with the regulations in order to gain access to medical cannabis. Patients in these situations may not be granted proper medical oversight or the education needed to increase their chances of success with medical cannabis treatment, which physicians should be aware of.

## Supplementary Information

**Additional file 1: Figure 1**. Patient Flow. **Figure 2**. Massachusetts Income Distributions. **Figure 3**. Colorado Income Distributions. **Figure 4**. Maine Income Distributions. **Figure 5**. Connecticut Income Distributions. **Figure 6**. Maryland Income Distributions.

## Data Availability

The datasets used for this article are not publicly available due to patients’ privacy. Data can be made available upon appropriate request to the authors.

## Abbreviations

CBD: Cannabidiol
IQR: Interquartile range
IRB: Institutional review board
PTSD: Post-traumatic stress disorder
SD: Standard deviation
THC: Δ-9-Tetrahydrocannabinol
US: United States
