# Supplementary material for: Comparing medical cannabis use in 5 US states: a retrospective database study
Source: J Cannabis Res. 2021 May 27;3:15. doi: 10.1186/s42238-021-00075-z (PMC8161659; doi:10.1186/s42238-021-00075-z)
Supplement: Supplementary file 1 — Additional file 1: Figure 1. Patient Flow. Figure 2. Massachusetts Income Distributions. Figure 3. Colorado Income Distributions. Figure 4. Maine Income Distributions. Figure 5. Connecticut Income Distributions. Figure 6. Maryland Income Distributions. [file 42238_2021_75_MOESM1_ESM.zip › CB2_2020_Supplemental Materials_Update Mar 2021_Clean.docx]

**Supplemental Materials**

**Figures**


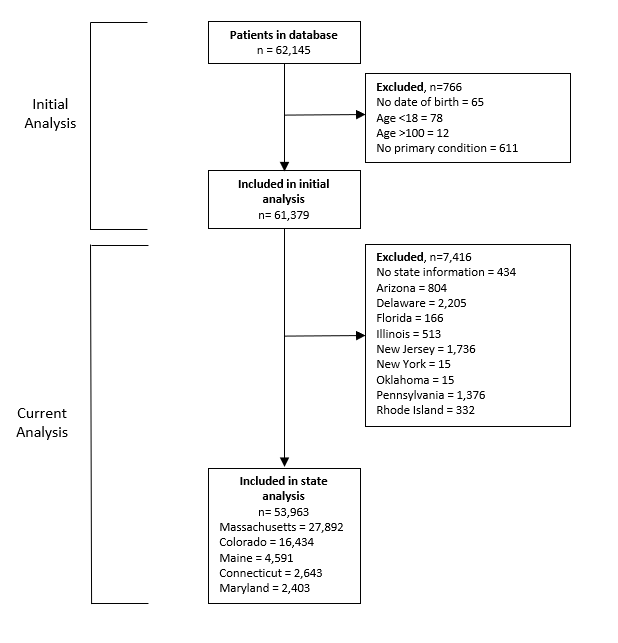


Figure 1. Patient Flow


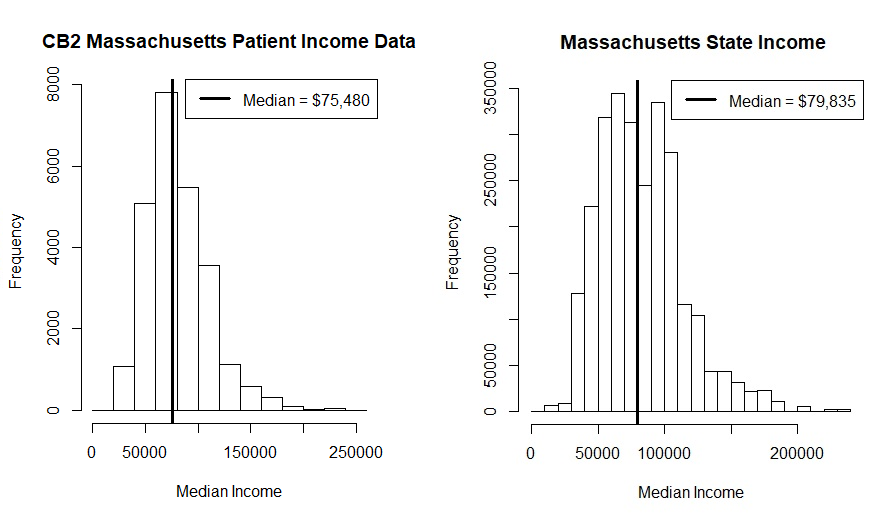


Figure 2. Massachusetts Income Distributions


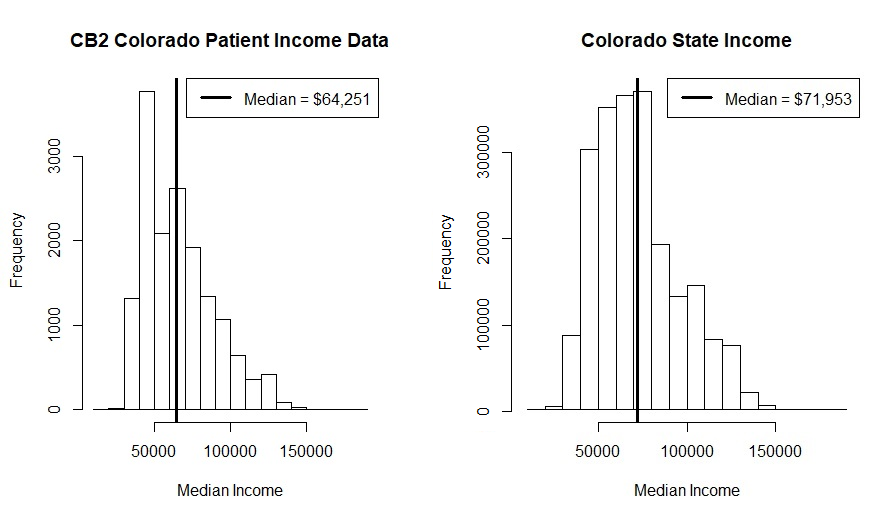


Figure 3. Colorado Income Distributions


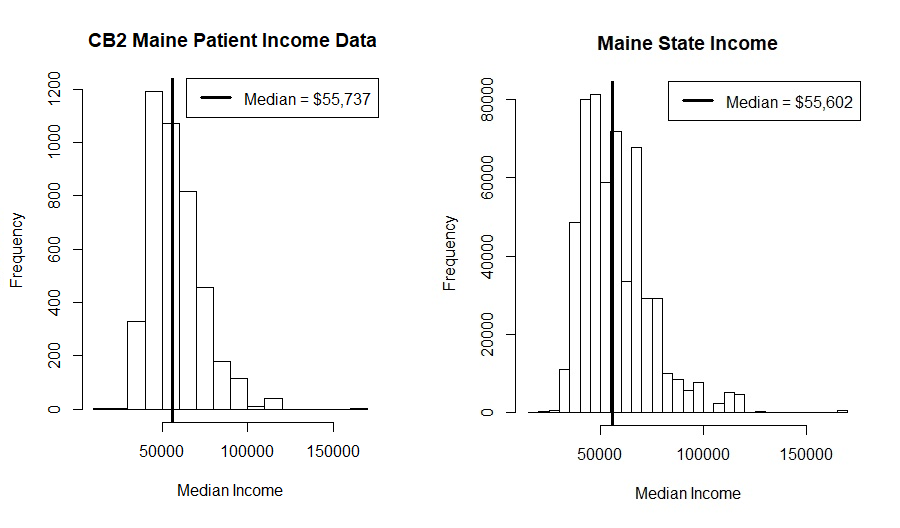


Figure 4. Maine Income Distributions


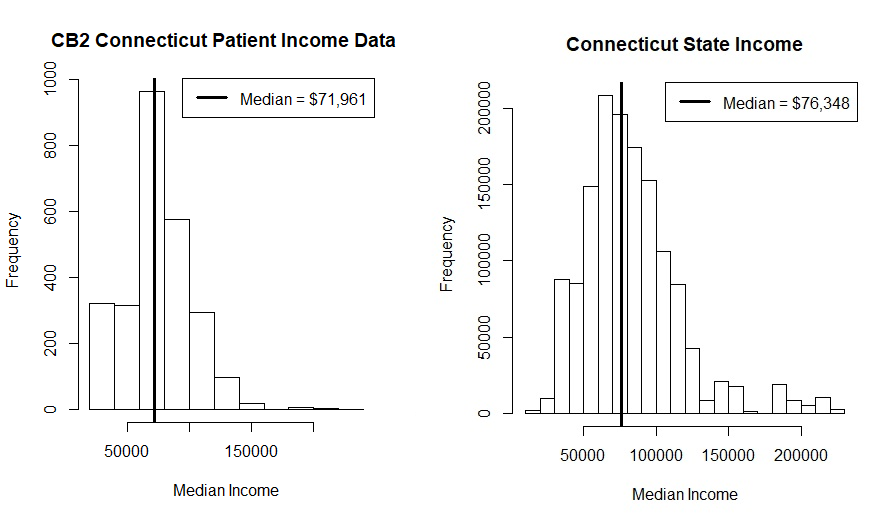
Figure 5. Connecticut Income Distributions


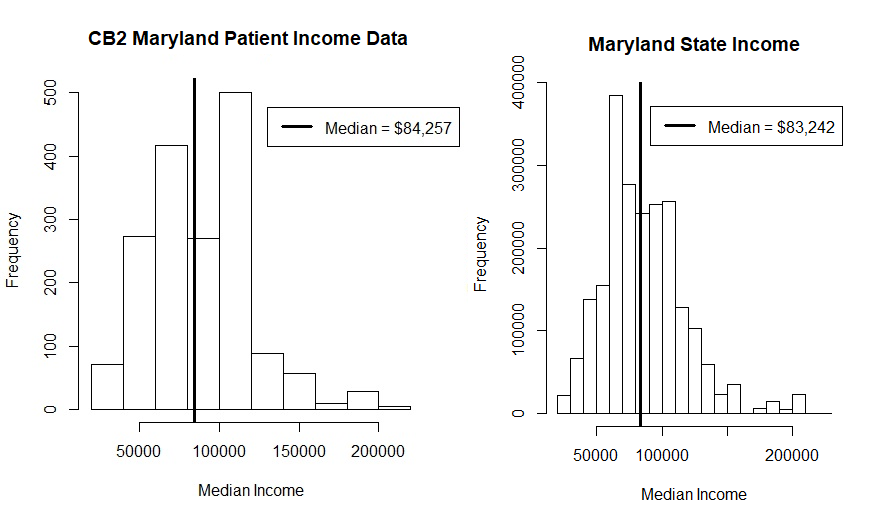


Figure 6. Maryland Income Distributions

**Figure Captions:**

Figure 1. Patient Flow

Figure 2. Massachusetts Income Distributions

Figure 3. Colorado Income Distributions

Figure 4. Maine Income Distributions

Figure 5. Connecticut Income Distributions

Figure 6. Maryland Income Distributions
