# Supplementary figures and images for: Comparing medical cannabis use in 5 US states: a retrospective database study
Source: J Cannabis Res. 2021 May 27;3:15. doi: 10.1186/s42238-021-00075-z (PMC8161659; doi:10.1186/s42238-021-00075-z)

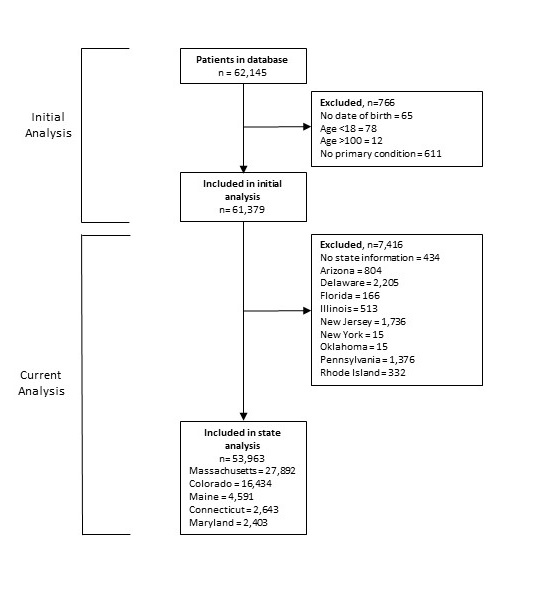

Supplement: Supplementary file 1 — Additional file 1: Figure 1. Patient Flow. Figure 2. Massachusetts Income Distributions. Figure 3. Colorado Income Distributions. Figure 4. Maine Income Distributions. Figure 5. Connecticut Income Distributions. Figure 6. Maryland Income Distributions. [file 42238_2021_75_MOESM1_ESM.zip › Supplemental_Figure 1.jpg]

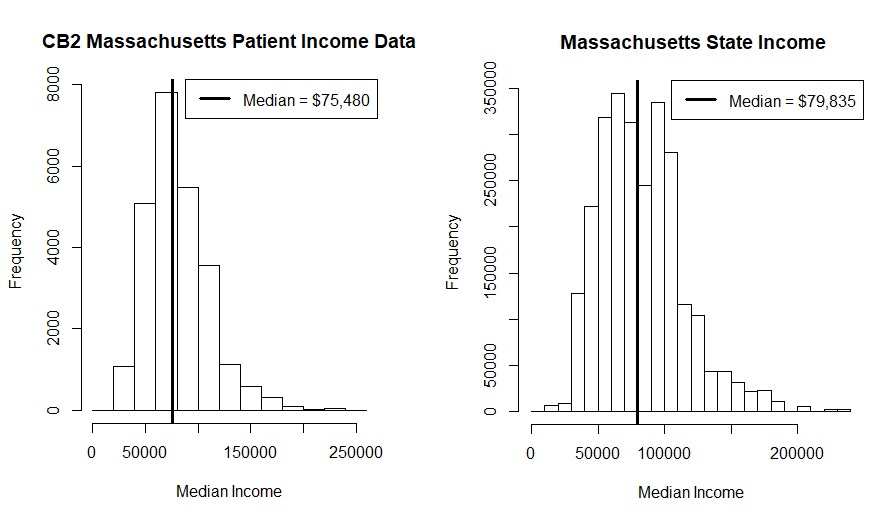

Supplement: Supplementary file 1 — Additional file 1: Figure 1. Patient Flow. Figure 2. Massachusetts Income Distributions. Figure 3. Colorado Income Distributions. Figure 4. Maine Income Distributions. Figure 5. Connecticut Income Distributions. Figure 6. Maryland Income Distributions. [file 42238_2021_75_MOESM1_ESM.zip › Supplemental_Figure 2_MA.jpg]

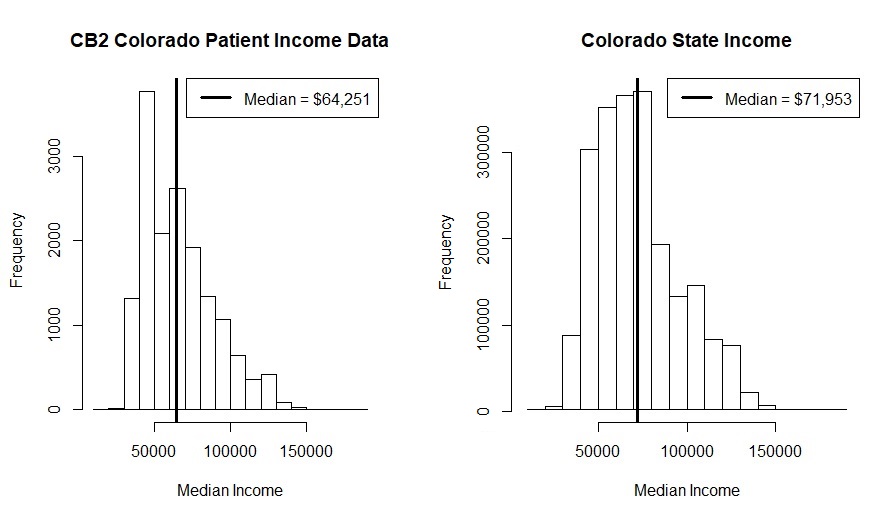

Supplement: Supplementary file 1 — Additional file 1: Figure 1. Patient Flow. Figure 2. Massachusetts Income Distributions. Figure 3. Colorado Income Distributions. Figure 4. Maine Income Distributions. Figure 5. Connecticut Income Distributions. Figure 6. Maryland Income Distributions. [file 42238_2021_75_MOESM1_ESM.zip › Supplemental_Figure 3_CO.jpg]

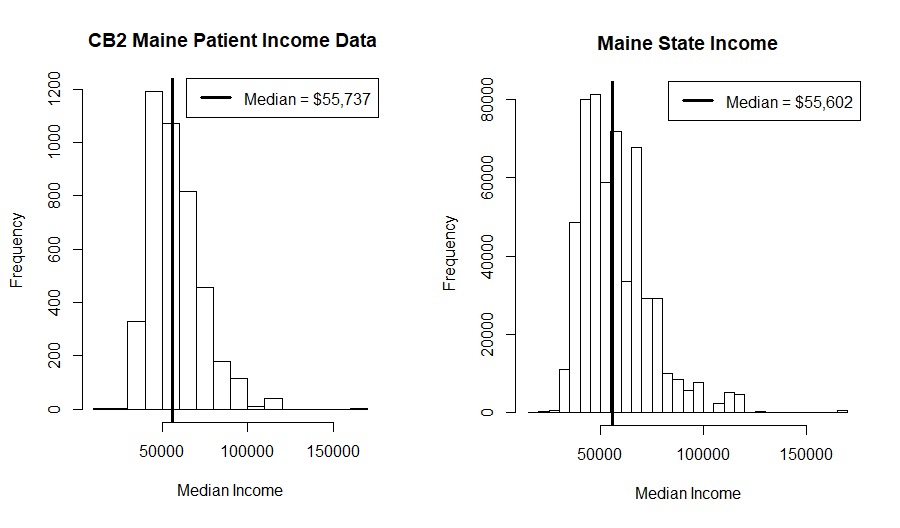

Supplement: Supplementary file 1 — Additional file 1: Figure 1. Patient Flow. Figure 2. Massachusetts Income Distributions. Figure 3. Colorado Income Distributions. Figure 4. Maine Income Distributions. Figure 5. Connecticut Income Distributions. Figure 6. Maryland Income Distributions. [file 42238_2021_75_MOESM1_ESM.zip › Supplemental_Figure 4_ME.jpg]

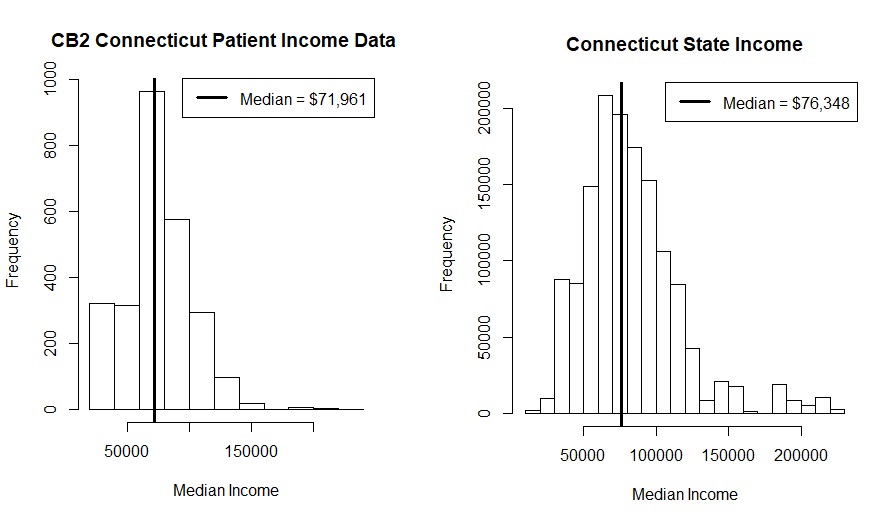

Supplement: Supplementary file 1 — Additional file 1: Figure 1. Patient Flow. Figure 2. Massachusetts Income Distributions. Figure 3. Colorado Income Distributions. Figure 4. Maine Income Distributions. Figure 5. Connecticut Income Distributions. Figure 6. Maryland Income Distributions. [file 42238_2021_75_MOESM1_ESM.zip › Supplemental_Figure 5_CT.jpg]

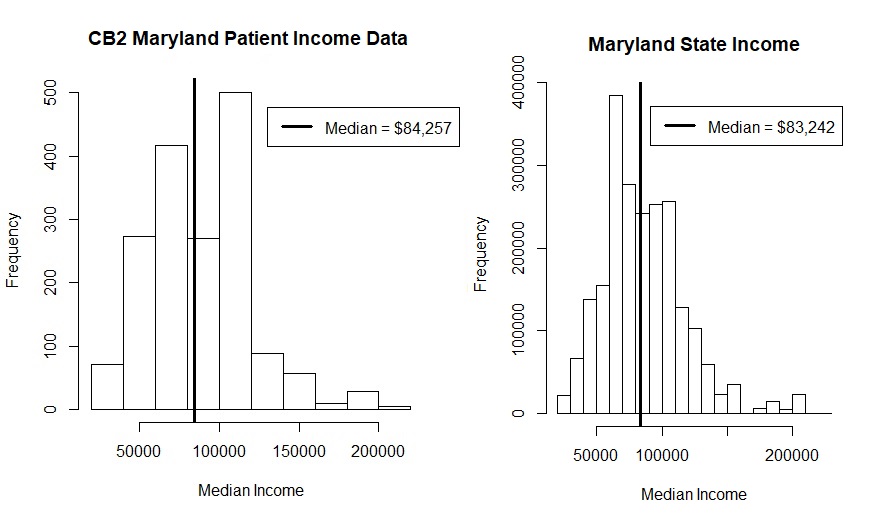

Supplement: Supplementary file 1 — Additional file 1: Figure 1. Patient Flow. Figure 2. Massachusetts Income Distributions. Figure 3. Colorado Income Distributions. Figure 4. Maine Income Distributions. Figure 5. Connecticut Income Distributions. Figure 6. Maryland Income Distributions. [file 42238_2021_75_MOESM1_ESM.zip › Supplemental_Figure 6_MD.jpg]
